# Utilization of dapsone and hemoglobin in the epithelial skin regeneration therapy of cutaneous loxoscelism: A case report and integrative literature review

**DOI:** 10.1590/1516-3180.2023.0151.04012023

**Published:** 2024-02-23

**Authors:** Omar Azuara-Antonio, Mario Isidoro Ortiz, Karla Daniela Jiménez-Oliver, Marco Castillo-Cabrera, Ana Karen Méndez-Salinas, Luz Hernández-Ramírez

**Affiliations:** I MD. Physician, Medical staff, emergency department, general hospital de Pachuca. Pachuca, Hidalgo, Mexico; Subject teacher, Academic Field of Medicine, Institute of Health Sciences, Universidad Autónoma del Estado de Hidalgo, Pachuca, Hidalgo, Mexico.; II MD, PhD. Professor, Department of Medicine, School of Health Sciences, Universidad Autónoma del Estado de Hidalgo. Pachuca, Hidalgo, Mexico.; III Medical student. Department of Medicine, School of Health Sciences, Universidad Autónoma del Estado de Hidalgo. Pachuca, Hidalgo, Mexico.; IV MD. Physician, Medical staff, emergency department, general hospital de Pachuca. Pachuca, Hidalgo, Mexico.; V MD. Physician, Medical staff, emergency department, general hospital de Pachuca. Pachuca, Hidalgo, Mexico.; VI MD. Physician, Medical staff, emergency department, general hospital de Pachuca. Pachuca, Hidalgo, Mexico.

**Keywords:** Loxosceles venom, Spider Bites, Case reports, Dapsone, Hemoglobins, Cutaneous loxoscelism, Loxosceles spider, Necrotic arachnidism

## Abstract

**BACKGROUND:**

*Loxosceles spp* are arthropods found worldwide. Its bite may produce cutaneous loxoscelism (necrotic or edematous) or cutaneous-visceral loxoscelism. Depending on their severity and location, cutaneous forms are managed with local cold application and systemic administration of antihistamines, corticosteroids, antibiotics, polymorphonuclear inhibitors, and analgesics.

**OBJECTIVE:**

This study aimed to report a case of cutaneous loxoscelism and to identify the main dermatological manifestations associated with the *Loxosceles spp* bite.

**DESIGN AND SETTING:**

This case report and literature review was conducted in a Mexican university.

**METHODS:**

A detailed report on the medical management of a patient with cutaneous loxoscelism treated at the emergency department of a public hospital was published. Scopus, PubMed, Web of Science, and Google Scholar databases were searched to identify articles reporting cutaneous loxoscelism. The following keywords were used during the database search: “loxoscelism” OR “spider bite,” OR “loxosceles” OR “loxosceles species” OR “loxosceles venom” OR “loxoscelism case report” AND “cutaneous” OR “dermonecrotic arachnidism.”

**RESULTS:**

A 62-year-old female patient with cutaneous loxoscelism was treated with systemic dapsone and local heparin spray. Eighteen studies with 22 clinical cases were included in this systematic review. Of the 22 patients, 12 (54.5%) were men. *L. rufescens* was the predominant spider species.

**CONCLUSIONS:**

The administration of dapsone and heparin for the management of cutaneous loxoscelism demonstrated success in this case, with no sequelae observed. In general, the literature review indicated favorable outcomes in patients treated with antimicrobials and corticosteroids, with continuous healing of skin lesions.

**SYSTEMATIC REVIEW REGISTRATION:**

PROSPERO ID CRD42023422424 (https://www.crd.york.ac.uk/prospero/display_record.php?ID=CRD42023422424).

## INTRODUCTION

Currently, more than 40,000 species of the order Araneae (grouped into approximately 4,205 genera and 128 families) have been identified. Spiders are distributed worldwide and cohabit with humans.^1,2^ Despite thousands of identified spider species, only a few are of clinical interest. Although some spider bites can cause severe or fatal poisoning in humans, most cause minor skin lesions. Only two spider bite syndromes are of clinical importance with a global distribution: latrodectism (caused by *Latrodectus* spp.) and loxoscelism (caused by *Loxosceles* spp.).^1-3^

Loxoscelism is the result of poisoning due to the injection of venom from *Loxosceles* spiders (solitary, recluse, fiddle-back, or brown spiders). Loxoscelism is classified as cutaneous, systemic, or viscerocutaneous.^1,4^ Cutaneous loxoscelism is the most prevalent form (85%), while viscerocutaneous is less common. Cutaneous loxoscelism can manifest as a flat erythematous plaque at the bite site and a sunken necrotic lesion of variable depth with benign evolution. Approximately 2–3 hours following a bite by *Loxosceles* spider, soft pain, erythema, and cyanosis may develop, with blisters or vesicles appearing on the skin. After several hours, the lesion became hemorrhagic and painful, accompanied by edema, erythema, ischemia, and thrombosis. An irregular area of ecchymosis or livedoid plaque with characteristic coloration (often resembling a bull’s eye or displaying red, white, and blue signs) emerged. Several days after the bite, the lesion may progress into a deep necrotic area, forming a dry necrotic eschar with sharp borders. Necrotic tissue sloughs off after a few weeks, leaving an ulcer with granulation tissue, which can take weeks or months to heal depending on the depth and extent of the lesion. Generally, no secondary infection occurs, and the lesion completely heals, leaving only a scar with variable characteristics.^1,4,5^ Patients with cutaneous loxoscelism may not present any systemic alterations or symptoms; however, generalized pruritus, arthralgia, headache, nausea, vomiting, and low-grade fever may occur.^1,3-5^ Edematous loxoscelism (5%), classified as a cutaneous type, is the most benign form. In such cases, the bite usually on the face results in extensive edema, erythema, and scant necrosis. By contrast, systemic loxoscelism (10%), in addition to the local dermonecrotic lesions with hemolysis, is accompanied by metabolic alterations, acute renal, pulmonary, and hematological damage (hemolytic anemia and coagulation disorders). It is lethal in approximately 15% of patients, primarily due to acute renal failure and disseminated intravascular coagulation.^1,3-5^

Loxoscelism is caused by the venom of *Loxosceles* spiders, with more than 100 species distributed across all continents. According to the findings of clinical case reports examining spider bites, *L. laeta, L. deserta, L. reclusa, L. gaucho, and L. intermedia* are the most important species.^1,3-6^ The venom of *Loxosceles* spiders contains sphingomyelinase-D (the main dermonecrotic and hemolytic factor), hyaluronidase, metalloproteases, 5 nucleotidases, collagenase, esterase, phospholipases, 5’ ribonucleotide phosphohydrolase, and alkaline phosphatase.^7,8^ The occurrence of hemolysis is mediated by complement activation and cytokine release, resulting in a clinical presentation resembling endotoxic shock. Inoculation with *Loxosceles* venom increases the concentrations of tumor necrosis factor, interleukins (ILs) 6 and 10, granulocyte-macrophage colony-stimulating factor, and nitric oxide.^3,7,8^ Edema, vascular endothelium thinning, inflammatory cell accumulation, vasodilation, coagulation, vascular wall degeneration, and hemorrhage occur in the bite area. These features are associated with vasculitis and contribute to tissue necrosis. Ceramides in the skin released by sphingomyelinase promote platelet adhesion and thrombus formation, which cause further alterations in the microcirculation. The inflammatory process and vasculitis with thrombus formation are the primary causes of local necrotic lesions. This leads to intravascular coagulation and areas of ischemia interspersed with hemorrhage, resulting in the distinctive marbled or livedoid plaque lesion characteristic of loxoscelism.^3,5,7,8^

The standard treatment of loxoscelism has not been established, and the approach depends on the type (cutaneous or systemic), time of the bite, visit to health services, clinical evolution of the patient, and probable complications. In cutaneous cases, local cold application, rest, elevation of the extremity if possible, and systemic pharmacotherapy with polymorphonuclear inhibitors (such as dapsone), and analgesics are recommended.^4,5,9^ The application of anti-loxosceles serum and hyperbaric oxygen may be indicated.^4,5,9^ In cases of systemic loxoscelism, it is crucial to closely monitor the renal and hepatic functions, correct severe hemolysis with blood products, administer bicarbonates to manage hemoglobinuria, and perform dialysis in the event of renal failure.^4,5,9,10^

## CASE REPORT

A 62-year-old female patient with a history of systemic arterial hypertension, managed pharmacologically for the past 5 years, received losartan 50 mg and metoprolol 50 mg twice daily. The patient experienced a myocardial infarction 4 years ago and a transient cerebrovascular event a month earlier, without significant sequelae from either event. The patient was treated with acetylsalicylic acid 100 mg and atorvastatin 40 mg once a day.

The patient arrived at the emergency department of our hospital with a spider bite on the inner region of the left thigh and severe burning pain. Upon examining a photograph of the spider, it was identified as an arachnid from the Sicariidae family and the genus *Loxosceles*.

A physical examination of the patient revealed a blood pressure of 113/74 mmHg, a heart rate of 90 beats per minute, a respiratory rate of 18 per minute, and a temperature of 36.2°C. Neurological assessment revealed the absence of alterations in the sensory or motor responses and showed no signs of focalization or frontalization. The laboratory tests yielded normal results according to the patient’s characteristics.

**Figure 1.**
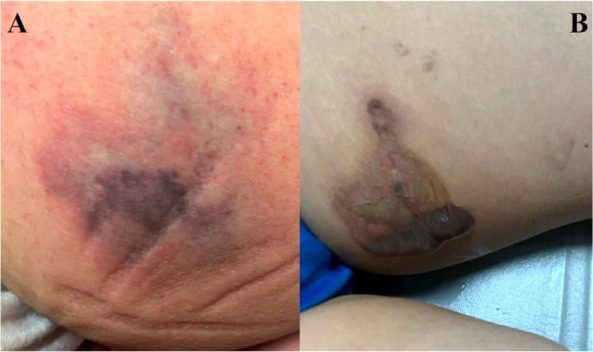
Skin lesions resulting from the bite of the *Loxosceles* spider. A: Lesion located in the inner region of the left thigh, measuring approximately 4 cm in diameter, exhibiting an undefined appearance with the presence of a livedoid plaque characterized by areas of erythema, ischemia, and necrosis, from the periphery to the center. B: Formation of vesicles with serohematic content, with irregular borders, delimited by an area of hyperpigmentation

Sixteen hours after the bite, the patient presented with an erythematous lesion measuring 2 cm in diameter with an indurated erythematous perilesional area or a bull’s-eye measuring 16 cm in diameter, with irregular edges and considerable pain upon palpation (Figure 1A). Treatment was initiated with dapsone 1 mg/kg body weight once a day, clindamycin 600 mg intravenously every 8 hours, and buprenorphine 150 *μ*g IV twice daily. After 24 hours, a blister approximately 12 cm in diameter developed on the lesion (Figure 1B).

**Figure 2.**
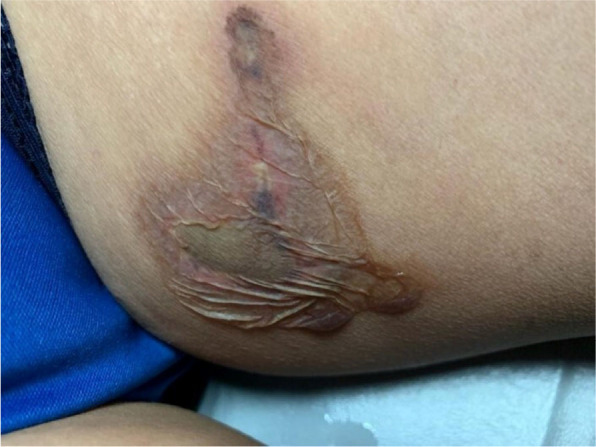
Lesion with irregular borders, necrotic background, delimited by an area of hyperpigmentation and erythema, showing exudate coming out of the vesicles

Twelve hours after the initiation of pharmacological treatment, the lesion became painless. The necrotic area diameter did not increase, while the perilesional induration decreased due to blister rupture. Additionally, a livedoid spot appeared (Figure 2).

Considering the favorable prognosis, the patient was discharged after 40 h of hospital stay. Dapsone 1 mg/kg body weight once daily for 7 days was prescribed as home treatment, and the patient was scheduled for follow-up at 7, 14, 21, and 28 days after the spider bite.

**Figure 3.**
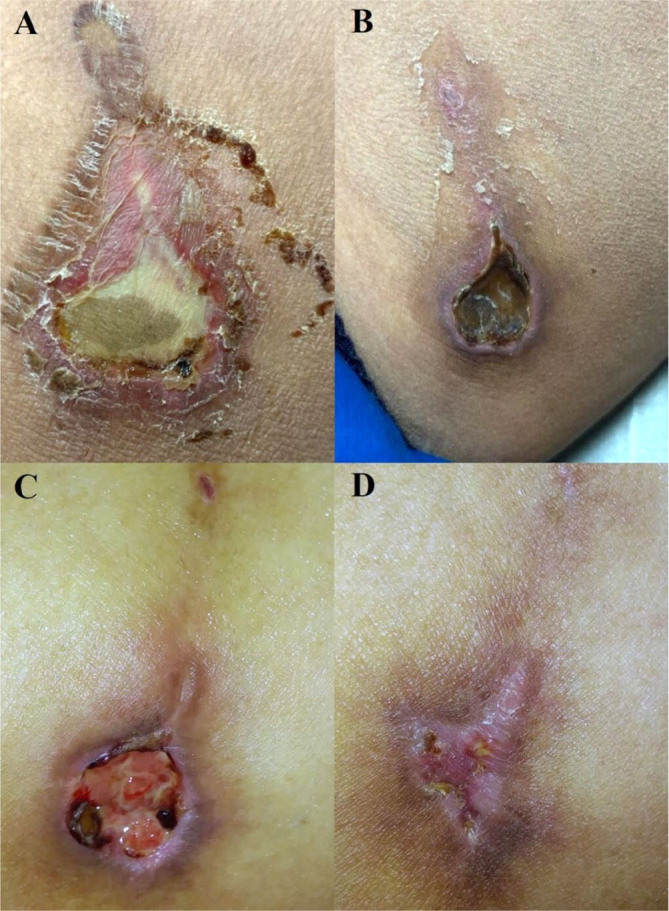
Lesion with irregular borders, delimited by a hyperpigmented area with scaly changes, erythema, and an area of central ischemia. A: Erythematous livedoid plaques with fine scaling and central necrotic eschar with accompanying scaling changes. B: Wound in the granulation phase, delimited by an area of hyperpigmentation. C and D: Lesions in the remodeling phase, with perilesional hyperpigmented area

During the 28-day appointment, debridement of the lesion was performed, followed by ulcer washing and topical application of Granulox^®^ (hemoglobin spray) (Figure 3A). Granulox^®^ was prescribed for at-home topical treatment every 8 hours for 7 days. Subsequently, the topical application of Granulox^®^ was indicated every 12 hours for 21 days and then every 24 hours for another 14 days. The patient returned for a follow-up examination of the lesion (Figure 3B-3D).

**Figure 4.**
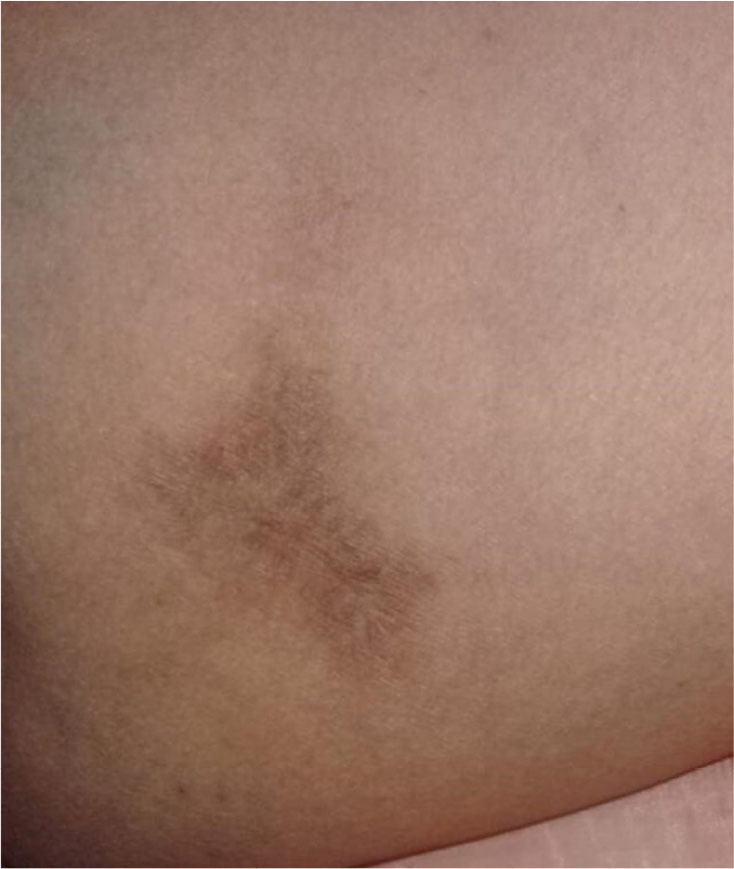
Complete remission of the lesion, discoloration, or formation of mild scar tissue

On day 70 following the spider bite, the patient underwent examination, revealing cutaneous epithelial regeneration that persisted until the lesion disappeared, leaving a hyperpigmented scar (Figure 4).

## METHODS

**Figure 5.**
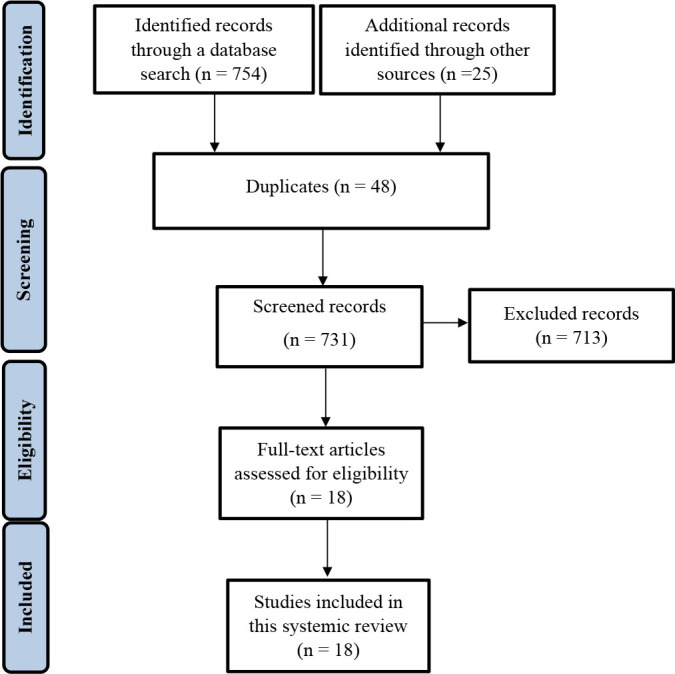
Preferred Reporting Item Guidelines for Systematic Reviews method included in this study

This systematic review was conducted in accordance with the guidelines of the Report of Paper for Systematic Reviews and Meta-Analyses (Figure 5).^11^ The study was developed and submitted to the International Prospective Register of Systematic Reviews. The Scopus, PubMed, Web of Science, and Google Scholar databases were searched to find articles reporting loxoscelism in Mexico. The following keywords were used during the database search: “loxoscelism” OR “spider bite,” OR “loxosceles” OR “loxosceles species” OR “loxosceles venom” OR “loxoscelism case report” AND “cutaneous” OR “dermonecrotic arachnidism.” A literature search was independently conducted by three reviewers until December 2022. The reference sections of relevant articles were manually searched to identify additional manuscripts. After the search, the manuscripts were imported into Mendeley Desktop 1.17.11 software (Glyph & Cog, LLC, London, UK) to eliminate duplicates.

Three independent reviewers evaluated the titles and abstracts of all publications. The following eligibility criteria were considered: (a) Systematic reviews, meta-analyses, case reports, and case series of patients of any sex and age presenting with local dermatologic manifestations derived from spider bites of the *Loxosceles* genus; (b) studies published in English, French, Portuguese, and Spanish languages; and (c) studies published between 1980 and 2022 were included in the analysis. Meanwhile, case reports involving patients with systemic signs or symptoms, focusing on cutaneous-visceral loxoscelism, with no available full text, and with insufficient data for the analysis were excluded. Articles that met the inclusion criteria were submitted for full-text evaluation. Any discrepancies in the decision-making process concerning the suitability of the included articles were resolved by consulting a fourth reviewer. Only publications that satisfied all the eligibility criteria were considered in the assessment.

The manuscript was systematically evaluated, and data were obtained from the studies after exhaustive evaluation. The following variables of interest were recorded on standardized worksheets: author, patient age, sex, country, location of the spider bite, size and type of lesion, local effects, time course of signs, spider species, and medical treatment. The Mix Methods Appraisal Tool (MMAT) was used for quality determination.^12^ The studies were analyzed by three reviewers in the following domains: clear research questions, adequate data collection, appropriate quantitative approach, appropriate methods to obtain the data, validation and interpretation of recorded data, appropriate statistical analysis, and interpretation. The fourth reviewer resolved any conflicts of interest. Using the MMAT, a score of 1 point was assigned for each quality marker, with a maximum total score of seven.

The results are summarized in Table 1. Qualitative and quantitative results were analyzed. Data analysis was performed using descriptive statistics and frequency measures (mean, standard deviation, minimum, median, maximum, frequency, and percentage).

**Table 1.** Summary of findings from the literature search, detailing location, size and type of lesion, local effects, time course of signs, species involved, treatment, age, gender, and country

Reference	Age in years	Sex	Country	spider species	Location of the spider bite	Size of the injury (cm^2^)	Kind of injury and local effects	Evolution time in weeks	Treatment and drugs
Farace et al.^13^ Case 1	75	M	Italy	Ru	Face	U	Necrotic ulcer, erythema, bull’s-eye (red, white, and blue sign), blister or vesicles, itchiness, and bleeding	5	Antibiotic, debridement and skin grafting, others
Farace et al.^13^ Case 2	50	M	Italy	Ru	Face	U	Necrotic ulcer, erythema, bull’s-eye (red, white, and blue sign), blister, and vesicles.	5	Antibiotic, debridement and skin grafting, others
Farace et al.^13^ Case 3	55	F	Italy	Ru	Thigh	60	Necrotic ulcer, erythema, bull’s-eye (red, white, and blue sign), pain, blister or vesicles, and bleeding	2	Antibiotic, corticosteroid, debridement and skin grafting, others
Hadanny et al.^14^ Case 1	30	M	Israel	Spp	Leg	2.3	Necrotic ulcer, erythema, swelling, and others	15	Antibiotic, hyperbaric chamber, others
Hadanny et al.^14^ Case 2	42	F	Israel	Spp	Hip	20	Necrotic ulcer, swelling, and others	14	Corticosteroid, hyperbaric chamber
Hadanny et al.^14^ Case 3	72	F	Israel	Spp	Medial malleolus	6.3	Necrotic ulcer, swelling, and others	12	Antibiotic, corticosteroid, hyperbaric chamber
Hillis et al.^15^	8	M	USA	Re	Upper and lower extremities	U	Erythema, bull’s-eye (red, white, and blue sign), blister or vesicles, and bleeding	4	Others
Miller et al.^16^	53	M	USA	Re	Arm	10	Necrotic ulcer, swelling, bull’s-eye (red, white, and blue sign), and pain	10	Antibiotic, hyperbaric chamber, debridement and skin grafting, others
Laack et al.^17^	46	M	USA	Re	Back	U	Erythema, bull’s-eye (red, white, and blue sign), and pain	3	Antibiotic, others
de Entrambasaguas et al.^18^	27	F	Spain	Ru	Face	U	Swelling, pain, and others	1	Antibiotic, corticosteroid, others
Bajin et al.^19^	69	F	Turkey	Ru	Face	U	Necrotic ulcer, erythema, and swelling	8	Antibiotic, corticosteroid
Yi et al.^20^	70	M	USA	Re	Leg	U	Necrotic ulcer, pain, and itchiness	7	Antibiotics, others
Hubiche et al.^21^	80	M	France	Ru	Arm	U	Erythema, bull’s-eye (red, white, and blue sign), and pain	1.8	NSAID
Molgó et al.^22^	30	M	Chile	La	Leg	U	Necrotic ulcer, erythema, and swelling	U	Debridement and skin grafting
Morales-Moreno et al.^23^	53	M	Spain	Ru	Thigh	20	Swelling, blister or vesicles, others, and itchiness	12	Antibiotic, NSAID
Guglielmetti et al.^24^	20	F	Chile	Spp	Thigh	50	Necrotic ulcer, erythema, bull’s-eye (red, white, and blue sign), blister, or vesicles	8	Antihistamine, corticosteroid, antibiotic, debridement and skin grafting, others
Trave et al.^25^	40	F	Italy	Ru	Thigh	U	Necrotic ulcer, erythema, bull’s-eye (red, white, and blue sign), blister, or vesicles	12	Antibiotic, corticosteroid, others
Esteban and Malmierca^26^	69	F	Spain	Ru	Face	4	Necrotic ulcer, swelling, bull’s-eye (red, white, and blue sign), pain, and fang marks	1	Antibiotic, corticosteroid, NSAID
Combi et al.^27^	79	F	Italy	Re	Breast	10	Necrotic ulcer, erythema, swelling, and itchiness	U	Antibiotic, hyperbaric chamber, others
Simões et al.^28^	23	M	Brazil	Spp	Genital organs	7	Necrotic ulcer, erythema, and pain	U	Antibiotic, NSAID, debridement and skin grafting, others
Luna-Muñoz et al.^29^	4	F	Peru	Spp	Face	U	Swelling and others	1.4	Antihistamine, Antibiotic, corticosteroid, antiloxosceles serum, others
Morales-Avalos et al.^30^	53	M	Mexico	Re	Face	U	Necrotic ulcer, erythema, swelling, and itchiness	3	Antibiotic, corticosteroid, debridement and skin grafting, others

F = female; M = male; U = undetermined; Re = *reclusa*; Ru = *rufescens*; La = laeta; Spp = species; NSAID = nonsteroidal anti-inflammatory drug

The Declaration of Helsinki was used to protect the privacy and confidentiality of the study patients. Formal written consent was sought from the patient for the utilization of photographic images depicting the evolution of the lesion resulting from the spider bite. Likewise, permission to publish the clinical case was obtained from the authorities of the hospital where the patient was treated.

## RESULTS

After the initial evaluation of the publications, 754 documents were obtained from the databases, and 25 additional records were identified by manual search. After removing the duplicates, 731 publications underwent screening based on their titles and abstracts. Of these, 713 did not meet the inclusion criteria and were excluded from the full-text review. A total of 18 articles were then subjected to a full-text review, all of which had sufficient data availability.^13-30^ For this systematic review, 18 full-text articles (2 investigating three cases and 16 reporting one clinical case) were included in the statistical analysis according to the inclusion, exclusion, and quality criteria (Figure 5).^13-30^ The selected studies were published between 1986 and 2022 and had cross-sectional designs. The quality ratings of the 18 case reports ranged from 6 to 7 according to the MMAT.^12^ Among these studies, 3 (16.7%) obtained a score of 6, while 15 (83.3%) obtained a score of 7.

In the analysis of 18 manuscripts included in the systematic review, 22 patients with cutaneous loxoscelism were reported to have no systemic or general involvement. Five (22.7%) case reports were conducted in Italy, 4 (18.2%) in the United States, 3 (13.6%) in Israel, 3 (13.6%) in Spain, and 7 in various countries (Table 1).^13-30^ A total of 22 patients were included in the analyses. Of the total patients, 12 (54.5%) were men. The patients had a mean (M) age (*±* standard deviation: SD) of 47.6 (22.8) years, with a minimum age of 4 years and a maximum age of 80 years.

According to previous reports, *Loxosceles rufescens* was involved in nine cases (40.9%), *Loxosceles reclusa* in six cases (27.3%), and *Loxosceles laeta* in one case (4.5%) (Table 1).^13-30^ In relation to the location of the spider bite, seven patients (31.8%) had a bite in the face, four (18.2%) in the thigh, three (13.6%) in the arm, three (13.6%) in the legs, and six in other regions of the body (Table 1).^13-30^ The primary types of lesions or local damages reported at the spider bite site were necrotic ulcer (n = 16), erythema (n = 14), inflammation (n = 12), bull’s-eye sign (n = 10), and blisters (n = 7) (Table 1). The primary treatments used were antibiotics (n = 18), corticosteroids (n = 10), analgesics (n = 4), and debridement (Table 1). The evolution time in weeks and the size of the injury in cm^2^ are listed in Table 1.^13-30^

## DISCUSSION

This case report focused on the clinical characteristics, treatment, and evolution of cutaneous loxoscelism. Here, we present the case of a 62-year-old female patient who was admitted to our emergency department due to cutaneous loxoscelism. In this case, comprehensive management of the patient involved systemic administration of dapsone for 7 days and local administration of hemoglobin for 45 days, achieving satisfactory improvement until remission.

In cutaneous loxoscelism, the intensity of the reaction depends on the amount of poison inoculated, the susceptibility of the patient to the components, and the time at which treatment is started. As an initial treatment, the application of a cold compress and the elevation of the affected part are recommended to minimize the spread of the inoculated venom and mitigate the inflammatory and metabolic cascades triggered by the venom at the lesion site.^1,4,5,9,31^ Likewise, systemic treatment with dapsone is recommended. It inhibits the release of the enzyme myeloperoxidase, blocks neutrophil adherence, and decreases the production of IL-8, prostaglandins, tumor necrosis factor-α, and histamine.^32^ Due to its antineutrophilic effect, dapsone is an effective treatment for skin lesions caused by the bite of the *Loxosceles* spider.^1,4,5,9,31,32^ Other lesion treatments have been used, such as silver sulfadiazine, chlorhexidine gluconate, sucrose therapy, surgical debridement, skin grafting, hyperbaric oxygen, and vacuum therapy.^5,9,33^ In our case report, a hemoglobin solution (Granulox® spray) was applied to the wound for several days until complete recovery, leaving only an acceptable scar and no sequelae. Granulox is a highly purified porcine hemoglobin fraction modified by carboxylation. It has been used as an auxiliary agent in the treatment of chronic wounds. The layer of hemoglobin applied to the wound transports and diffuses oxygen to the hypoxic surface tissues and helps maintain moisture in the wound to facilitate optimal healing.^6^ To our knowledge, this case report is the first to document the use of a hemoglobin spray for the successful treatment of a *Loxosceles* spider bite wound.

Our literature review revealed a scarcity of studies and case reports on cutaneous loxoscelism without systemic symptoms. Loxoscelism can be classified as cutaneous or dermonecrotic and systemic or cutaneous-visceral.^5^ Another classification system stratifies it according to severity: mild or grade 1 (erythema, punctum, no necrosis, and pruritus), moderate or grade 2 (erythema, mild edema, vesicle, pain, and necrosis *≤*1 cm^2^), severe or grade 3 (erythema, edema, hemorrhagic bullae, pain, ulcer, and necrosis >1 cm^2^), and systemic (skin lesions grade I, II, or II, plus rash, fever, hemolysis, thrombocytopenia, myalgia, headache, and disseminated intravascular coagulation).^31^ In a systematic review of 120 case reports of loxoscelism, 89 (74%) cases were classified as cutaneous, of which 22 (25%) only had local symptoms.^5^

Several studies have explored the correlation between the presence and severity of loxoscelism and the patient’s characteristics, such as age, sex, the site of the bite, comorbidities, and the immune system status.^3-5,31,34^ Some reports have suggested an elevated risk of more severe loxoscelism in children, adolescents, and older adults.^34^ However, other authors have not identified a specific age with a higher prevalence.^3-5,31,34^ Our review aligns with the latter. Although the average age of the 22 patients was 40 years, loxoscelism was reported in all age groups. Our results in terms of age are consistent with the data reported from 200 patients with loxoscelism in Chile.^10^ In terms of sex, some studies reported a higher prevalence in women, primarily due to their increased involvement in domestic activities.^5^ However, other studies did not observe a significant difference in the prevalence of loxoscelism between men and women,^36^ which was also observed in 54.5% of men in our study. According to literature reports, the upper or lower extremities, face, neck, and thorax are the most common sites of *Loxosceles* spider bites.^10^ This greater predilection of bite sites occurs accidentally or randomly. In the present study, the main sites of the lesions were the face and extremities. Consequently, individuals should inspect the objects that they come into contact with, shake clothes and beddings before use, and constantly clean and rearrange the furniture.

Globally, 143 species of *Loxosceles* spiders have been documented, with approximately 122 species found in America. Among these, only the *L. reclusa, L. deserta, L. arizonica, L. rufescens, L. laeta, L. Gaucho,* and *L. intermedia* species are of great clinical interest.^8,37,38^ In Mexico (which is divided into 32 federal entities or states), 28% of the 143 identified species are found. Noteworthy species with broad distribution and medical importance in Mexico were *L. deserta, L. boneti, L. reclusa*, *and L. arizonica.* In the state of Hidalgo (one of the 32 states of Mexico and where the patient in the case report resides), four *Loxosceles* species (*L. jaca, L. nahuana, L. Tenango, and L. tolantongo*) have been identified.^2,35^ In terms of the toxicity level of *Loxosceles* venom, variations in enzymatic constituents and substrate preferences contribute to different lethal effects. For instance, the lethal dose required by each species is higher for *L. laeta* (1.45 mg/kg) and lower for *L. similis* (0.32 mg/kg). Additionally, female spiders produce a greater amount of venom owing to the greater weight and size of their bodies, enhancing the venom’s impact.^39-41^ In the literature review of our study, the main species identified were *L. rufescens* and *L. reclusa*. In our case report, the specific specie of *Loxosceles* spiders was not identified. The identification of the spider species is crucial for generating accurate statistics, since our country lacks precise records of poisonings related to loxoscelism (accounting for only 5% of cases). Consequently, many cases of loxoscelism may be overlooked, leading to misdiagnosis. The lesions are often mistaken for other conditions, including necrotizing fasciitis, deep vein thrombosis, ulcers due to diabetes, infections of bacterial origin, cutaneous anthrax, erythema multiform, and lymphomas.^42^

With regard to the presentation of skin lesions in the 22 patients included in our literature review, erythema was observed in 63%, aligning with the findings of other systematic reviews reporting percentages between 68% and 72%.^43,44^ Another investigation reported a lower occurrence, with only 17% of patients presenting with erythema, of whom 8.5% were specifically associated with cutaneous loxoscelism.^10^ This variation could be attributed to factors such as the amount of venom inoculated, the species of the spider, and the individual immune response of the patients.^10^ In more than half of the patients in our sample, their skin lesion progressed into a necrotic lesion, a prevalent characteristic consistent with other reviews reporting percentages equal to or exceeding 50%.^43-45^ The formation of vesicles, inflammation, and the characteristic bull’s-eye sign were also frequently observed in the patients included in our review, consistent with the findings of other studies.^3,5,31,43,45^

As previously reported, the initial treatment of cutaneous loxoscelism centers on decreasing the distribution of venom through local cold application and elevation of the affected site, with the goal of limiting skin injury for prompt and effective recovery. The use of dapsone is recommended to reduce inflammatory reactions and skin injury.^4,5,9^ Other systemic treatments include antivenom, corticosteroids, hyperbaric oxygen, and antihistamines. In our literature review, the primary treatments administered were antibiotics and corticosteroids (Table 1),^13-30^ and only one patient received dapsone.^24^ In this particular case, the necrotic process was stabilized with dapsone. However, ulcer healing was delayed.^24^ On the contrary, surgical debridement and skin grafting were also performed in eight (36.4%) patients in our review (Table 1). This latter treatment is rarely recommended due to the potential for increased damage.^4,5,9^

The present study has several limitations. One limitation is the scarcity of information and statistics available on case reports of cutaneous loxoscelism without systemic manifestations. Many of these reports, being incomplete or of poor quality, lack the necessary data to construct a comprehensive clinical description, affecting the formulation of the conclusions. Variations in the type and severity of the lesion are influenced by the amount of venom inoculated, the patient’s time of arrival for medical evaluation, and the initiation of medical treatment post-bite. Currently, no method has been established to measure the amount of venom inoculated, which creates another limitation in published studies.^44^ In only 5% of patients, it is possible to identify the arachnids species involved. In our clinical case, the spider specie could not be identified.^4,5,9^ This limitation hinders the generation of accurate statistics and the provision of an appropriate diagnosis.^42^ Currently, no systematic reviews have evaluated patients with cutaneous loxoscelism without local manifestations. Therefore, healthcare professionals must identify the signs and symptoms to facilitate the correct classification of cases.

## CONCLUSION

The management of patients with cutaneous loxoscelism using dapsone and heparin proved favorable, with no sequelae. Reports on cutaneous loxoscelism without systemic manifestations in the global literature remain scarce. Generally, cases of cutaneous loxoscelism without systemic involvement, as reported in the literature, have shown favorable outcomes with the administration of antimicrobials and corticosteroids, facilitating the continuous healing of the skin lesion.
